# Anterior cervical osteophyte-related dysphagia in a long-term growth hormone user: a case report

**DOI:** 10.3389/fsurg.2026.1859548

**Published:** 2026-07-03

**Authors:** Sui-Ling Perez, Lynette Martinez, Michael Rosselli, Rakesh Ravikumaran Nair

**Affiliations:** 1 Florida International University Herbert Wertheim College of Medicine, Miami, FL, United States; 2 South Miami Walk-in Orthopedics and Sports Medicine, Miami, FL, United States

**Keywords:** anterior cervical osteophyte, cervical spondylosis, dish, dysphagia, growth hormone, IGF-1, sports injury

## Abstract

**Background:**

Anterior cervical osteophytes can compress the esophagus, producing dysphagia. Although common on imaging in older adults, symptomatic osteophytic dysphagia is underdiagnosed, and the role of systemic anabolic factors in osteophyte growth is poorly characterized.

**Case description:**

A 62-year-old male ex-military recreational bodybuilder presented with cervicalgia and left shoulder pain. Cervical radiographs and MRI demonstrated multilevel degenerative changes including a prominent anterior osteophyte at C3-C4 with esophageal compression. On directed questioning, he reported intermittent dysphagia with larger food boluses. He reported a cervical injury during jiu-jitsu training in 2001–2002 and disclosed approximately 20 years of patient-reported exogenous growth hormone (GH) and GH secretagogue use, including cyclical somatropin, sermorelin/GHRP-6 combination therapy, and ongoing nightly somatropin. No objective swallowing evaluation was performed given the mild, intermittent nature of symptoms.

**Conclusion:**

This case raises the hypothesis that chronic GH axis stimulation, in combination with remote cervical trauma, may contribute to clinically significant osteophyte formation. Conservative management was initiated with dietary modification and counseling regarding GH cessation. Clinicians should screen for dysphagia in patients with cervical spondylosis and inquire about anabolic substance use as a potentially relevant history.

## Introduction

Anterior cervical osteophytes (ACOs) are bony spurs arising from the anterior vertebral bodies, most commonly due to degenerative spondylosis, diffuse idiopathic skeletal hyperostosis (DISH), or post-traumatic remodeling (1–3). Their prevalence increases with age, estimated at 20%–30% in individuals over 60, though most remain asymptomatic (4, 5). Symptomatic ACOs can compress the esophagus, pharynx, and airway, producing dysphagia, dysphonia, globus sensation, or aspiration, a constellation first described by Mosher in 1926 (6). At C3-C6, where the hypopharynx transitions to the cervical esophagus, the esophagus is particularly vulnerable to compression (7, 8).

The pathogenesis of exuberant ACO formation is multifactorial. Cervical injuries are described in contact and combat sports (9–11), and traumatic disruption of the anterior longitudinal ligament, periosteum, and vertebral endplates may contribute to heterotopic bone formation (12). Exogenous growth hormone (GH) and GH secretagogues stimulate the GH/IGF-1 axis, exerting skeletal anabolic effects that resemble the arthropathy of acromegaly (13–17). Despite their widespread use among recreational athletes and military populations, the potential contribution of these agents to cervical osteophyte formation has not been characterized.

We present a case of osteophytic dysphagia in a patient with remote cervical trauma and prolonged patient-reported GH and secretagogue use. Through this case, we explore the hypothesis that chronic GH axis stimulation may represent a modifiable contributor to osteophyte growth.

## Case description

### Patient background

A 62-year-old male presented to an outpatient orthopedic and sports medicine clinic on January 29, 2025, with cervicalgia and follow-up evaluation of left shoulder pain previously assessed in November 2024. He was an ex-military veteran with a recreational bodybuilding background. Past medical history included hypertension, chronic low back pain with lumbar radiculopathy, left glenohumeral and acromioclavicular osteoarthritis, left rotator cuff tendinitis, and a nontraumatic left proximal biceps tendon rupture. He denied alcohol, tobacco, and recreational drug use.

The patient reported a cervical hyperextension/hyperflexion injury during Brazilian jiu-jitsu training in approximately 2001–2002. He also reported approximately two decades of exogenous GH and GH secretagogue use, summarized in the timeline (Table 1). This exposure history is entirely patient-reported and was not verified through prescriptions, pharmacy records, or laboratory data. The patient suspected that his prolonged GH use may have contributed to the cervical osteophyte and to the skeletal changes necessitating his ankle arthroscopy in 2021.

**Table 1:** Timeline of key events. GH and secretagogue exposure history is entirely patient-reported and was not independently verified through prescriptions, pharmacy records, or laboratory documentation.

Date	Event	Details
∼2001–2002	Cervical trauma	Patient-reported hyperextension/hyperflexion injury during Brazilian jiu-jitsu training.
∼2005–2025	GH/secretagogue use (patient-reported)	Cyclical somatropin (∼2005–2016, 0.4 mg SC 1–2× daily, up to 6 days/week); sermorelin + GHRP-6 (∼2016–2019+, 400 mcg SC up to 3× daily); ongoing somatropin (0.4 mg SC nightly, 5 nights/week at presentation). Cumulative ∼20 years.
Jan 29, 2025	Cervicalgia presentation and dysphagia disclosure	Cervical x-rays: multilevel degeneration, prominent anterior osteophyte at C3-C4. On directed questioning, patient reported intermittent dysphagia with larger food boluses.
Feb 2025	Cervical MRI	Multilevel degenerative changes C2-C7 with moderate-to-severe stenosis. Prominent anterior osteophyte at C3-C4 with esophageal compression.
Feb 2025	Conservative management initiated	Dietary modification, NSAIDs as needed, home exercise program. Counseled on potential role of GH use; GH cessation and endocrine referral recommended.
Sep 2025	Chest wall evaluation	Chest x-rays showed multiple abnormal bony projections along the sternum and diffuse degenerative thoracic changes, suggestive of systemic hyperostosis.

### Clinical findings

At the January 2025 visit, the patient described acute-on-chronic cervicalgia with left posterior shoulder discomfort managed with occasional ibuprofen and a home exercise program for two months without significant improvement.

Examination revealed a well-developed male with normal gait. Cervical range of motion showed normal flexion, extension limited to 30 degrees, right rotation 70 degrees, left rotation 60 degrees, and bilaterally limited lateral bending. Inspection showed normal alignment without tenderness or masses. Cervical compression and bilateral Spurling tests were negative.

Neurologic examination demonstrated 5/5 strength bilaterally in the upper extremities with intact dermatomal sensation and normal reflexes. Hoffmann sign, Babinski sign, clonus, and tandem gait were not formally documented in the clinical record. The patient denied bowel or bladder dysfunction, hand clumsiness, fine motor impairment, and balance problems. No hyperreflexia or spasticity was noted on examination. While the neurologic examination did not reveal overt myelopathic signs, we acknowledge that a comprehensive myelopathy assessment including Hoffmann sign, Babinski sign, clonus, and tandem gait testing would have strengthened the evaluation, particularly given the moderate-to-severe multilevel cervical stenosis identified on MRI. This represents a limitation of the clinical encounter.

Left shoulder examination showed decreased forward flexion and abduction (165 degrees with pain), decreased external rotation, 4/5 strength in forward flexion, abduction, and external rotation, and positive Apprehension, Empty Can, O'Brien, and Speed tests.

## Timeline

Table 1 presents the key events across the episode of care.

## Diagnostic assessment

AP and lateral cervical spine radiographs (January 29, 2025) demonstrated loss of cervical lordosis, multilevel facet joint hypertrophy, disc space narrowing, and osteophytes. The lateral view showed a prominent anterior osteophyte at C3-C4 with potential esophageal compression (Figure 1).

**Figure 1:**
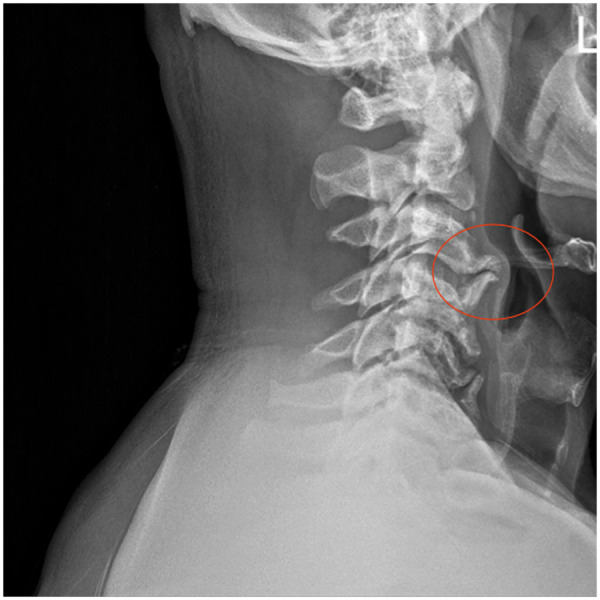
Lateral cervical spine radiograph demonstrating the prominent anterior osteophyte at C3-C4 (circle) projecting anteriorly with potential esophageal compression.

MRI of the cervical spine without contrast (February 2025) revealed multilevel degenerative changes with straightening of the cervical lordosis. The key finding was a prominent anterior osteophyte at C3-C4 with esophageal compression. Additional findings included diffuse disc bulges at C2-C3 and C3-C4 with moderate-to-severe central canal stenosis, and disc/osteophytic ridges at C4-C5 through C6-C7 with severe central canal and neural foraminal stenosis. C7-T1 was patent (Figure 2).

**Figure 2:**
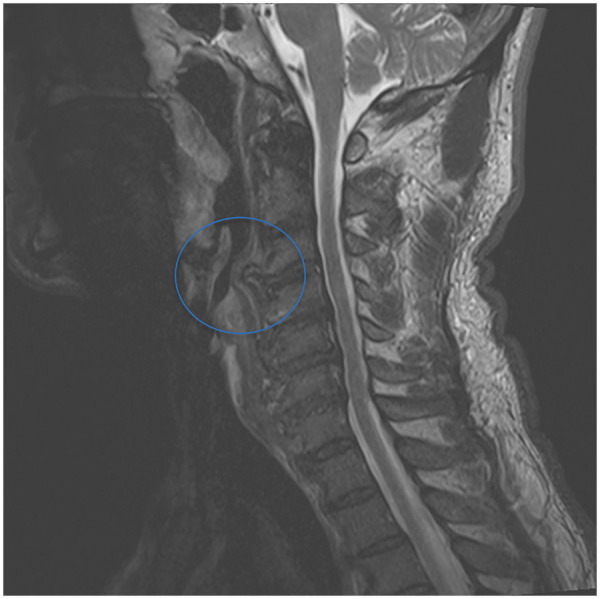
Sagittal T2-weighted MRI of the cervical spine demonstrating multilevel degenerative disc disease, central canal stenosis, and a prominent anterior osteophyte at C3-C4 (circle) abutting the esophagus.

### Dysphagia assessment

Upon directed questioning about the C3-C4 osteophyte, the patient disclosed occasional difficulty swallowing, particularly with larger food boluses and during nasal congestion. He had not previously reported this symptom. The dysphagia was intermittent without aspiration, weight loss, or dietary restriction. A barium swallow or videofluoroscopic swallowing study (VFSS) was not performed given the mild, intermittent nature of symptoms. The absence of objective swallowing evaluation is a limitation of this case and is discussed further in the Limitations section. Should dysphagia progress, formal swallowing studies would be indicated.

### Diagnostic reasoning

Radiographic and MRI correlation confirmed a clinically significant anterior osteophyte at C3-C4 with esophageal compression in the context of multilevel cervical spondylosis. The severity of degenerative changes spanning C2-C7 in a 62-year-old patient raises the question of contributing systemic factors, though age-related spondylosis alone could account for these findings.

The differential diagnosis for exuberant anterior osteophyte formation includes degenerative spondylosis, post-traumatic remodeling, and DISH. The patient's self-reported remote cervical trauma and prolonged GH exposure represent potential contributing factors, though their individual or combined roles cannot be established from this case alone. The September 2025 chest x-ray findings of multiple abnormal bony projections along the sternum and diffuse degenerative thoracic changes are suggestive of systemic hyperostosis but have not been formally evaluated against the Resnick criteria for DISH (18), and this remains an unconfirmed diagnostic consideration.

## Therapeutic intervention and follow-up

Given the mild, intermittent dysphagia without aspiration, conservative management was selected: dietary modification (smaller boluses, thorough mastication, upright positioning), as-needed nonsteroidal anti-inflammatory medications, and continuation of the home cervical exercise program. The patient declined a Medrol dose pack.

Counseling was provided regarding the hypothetical contribution of long-term GH and secretagogue use to skeletal overgrowth, and cessation of exogenous GH was recommended along with referral for endocrine evaluation and baseline IGF-1 measurement. The patient was informed that surgical osteophytectomy via an anterior cervical approach remains an option should symptoms progress.

At most recent follow-up, the patient remained on conservative management with stable, intermittent symptoms and no progression to aspiration, weight loss, or dietary restriction.

## Discussion

This case illustrates three clinically relevant points: anterior cervical osteophytes as an underrecognized cause of dysphagia, the potential role of remote cervical trauma in osteophyte initiation, and the hypothesis that chronic GH/IGF-1 axis stimulation may contribute to accelerated skeletal overgrowth.

### Osteophytic dysphagia

Dysphagia secondary to ACOs is an underdiagnosed entity. The esophagus lies in direct apposition to the prevertebral fascia, making it vulnerable to compression (7, 8). Symptoms range from mild intermittent dysphagia to complete obstruction, aspiration pneumonia, and airway compromise (19, 20, 31, 33). Beyond direct mechanical compression, local inflammation and disturbance of the swallowing mechanism, including cricopharyngeal dysfunction, have been described (21–23). Diagnosis typically involves lateral cervical radiography, barium swallow or VFSS, and cross-sectional imaging (8, 24). In our patient, the diagnosis was supported by imaging correlation between the C3-C4 osteophyte and the patient's symptoms, though the absence of a formal swallowing study limits the strength of this conclusion.

### Cervical trauma and osteophyte pathogenesis

Cervical trauma is a recognized initiator of accelerated spondylotic change. Hyperextension/hyperflexion injuries can disrupt the anterior longitudinal ligament, periosteum, and endplates, which has been proposed to stimulate heterotopic bone formation (9–12). Cervical trauma has been proposed as an initiator of accelerated spondylotic change at injured levels (25, 26). Our patient reported a jiu-jitsu injury approximately two decades before the current findings. While this is consistent with the recognized potential for cervical injury in grappling and combat sports (9, 11) and in athletes more broadly (27), there is no contemporaneous imaging, acute injury documentation, or serial radiologic evidence to confirm that the current osteophyte is directly attributable to this reported injury. The trauma history is presented as a plausible contributing factor rather than a confirmed etiology.

### Growth hormone and skeletal overgrowth: a hypothesis

Growth hormone exerts skeletal effects primarily through IGF-1, stimulating chondrocyte proliferation, endochondral ossification, and periosteal bone apposition (16). In acromegaly, characteristic manifestations include osteophyte formation, joint space widening, and diffuse hyperostosis (13, 14, 17). Exogenous GH, even at supraphysiologic doses, has been associated with features resembling mild acromegalic skeletal disease (15, 28). GH secretagogues such as sermorelin and GHRP-6 stimulate pulsatile endogenous GH release with downstream IGF-1 elevation.

Our patient reported approximately 20 years of GH axis stimulation through both direct somatropin administration and secretagogue-mediated amplification. We hypothesize that this prolonged exposure may have contributed to the exuberance of osteophyte formation, potentially in synergy with the reported remote cervical trauma. However, it is important to emphasize that a causal relationship cannot be established from a single case. The GH exposure history is self-reported and unverified, contemporaneous IGF-1 levels are unavailable, and the observed skeletal changes could be attributable to age-related spondylosis, post-traumatic remodeling, or other factors independent of GH use. The association is presented as a biologically plausible hypothesis warranting further investigation.

### DISH as a differential consideration

The multilevel anterior osteophyte formation and sternal bony projections raise the possibility of DISH (Forestier disease). DISH is characterized by flowing anterior longitudinal ligament ossification spanning at least four contiguous vertebral levels with relative disc space preservation and absence of apophyseal joint ankylosis (18). Elevated GH and IGF-1 have been implicated in DISH pathogenesis (29, 30, 32). However, DISH has not been formally confirmed in this patient, as dedicated thoracolumbar imaging and assessment against the Resnick criteria were not performed. We therefore describe the skeletal findings as suggestive of systemic hyperostosis rather than confirmed DISH, and acknowledge this as a limitation.

### Strengths and limitations

Strengths of this report include detailed documentation of a prolonged GH and secretagogue exposure history spanning two decades, multimodal imaging confirmation of osteophytic esophageal compression, and integration of a remote sports injury history that is rarely captured in cervical spondylosis evaluations. The case highlights the clinical importance of directed questioning about both dysphagia and anabolic substance use.

Several limitations should be noted. First, this is a single case, and causality between GH exposure and osteophyte formation cannot be established. Second, the GH and secretagogue exposure history is entirely patient-reported and was not verified through prescriptions, pharmacy records, or laboratory documentation. Third, contemporaneous serum IGF-1 measurements were not obtained, precluding correlation between GH exposure levels and skeletal changes. Fourth, a barium swallow or VFSS was not performed; while the imaging findings and symptom pattern are consistent with osteophytic dysphagia, the absence of objective swallowing evaluation limits the diagnostic certainty. Fifth, DISH was not formally evaluated with dedicated thoracolumbar imaging or the Resnick criteria. Sixth, a comprehensive myelopathy-focused neurologic assessment (including Hoffmann sign, Babinski sign, clonus, and tandem gait) was not documented, which is a notable gap given the moderate-to-severe multilevel stenosis identified on MRI. Finally, genetic and metabolic contributors to skeletal hyperostosis were not screened.

## Take-away lessons

Anterior cervical osteophytes should be considered in the differential diagnosis of dysphagia, and clinicians should routinely ask about swallowing difficulties in patients with cervical spondylosis. A thorough history should include prior cervical trauma and anabolic substance use. Long-term exogenous GH and secretagogue use may represent a biologically plausible, potentially modifiable contributor to skeletal overgrowth, though this hypothesis requires validation in prospective studies.

## Patient perspective

The patient acknowledged the potential contribution of his long-term growth hormone and secretagogue use to skeletal overgrowth and the cervical osteophyte. He expressed concern about the possible systemic effects of prolonged GH exposure and stated his intent to discontinue these agents. He agreed to proceed with conservative management, including dietary modification and the home exercise program, and to follow up should dysphagia worsen or new symptoms develop. He consented to the publication of this report to contribute to awareness of this potential association.

## Data Availability

The original contributions presented in the study are included in the article/Supplementary Material, further inquiries can be directed to the corresponding authors.
